# A new glance at the chemosphere of macroalgal–bacterial interactions: In situ profiling of metabolites in symbiosis by mass spectrometry

**DOI:** 10.3762/bjoc.17.91

**Published:** 2021-05-19

**Authors:** Marine Vallet, Filip Kaftan, Veit Grabe, Fatemeh Ghaderiardakani, Simona Fenizia, Aleš Svatoš, Georg Pohnert, Thomas Wichard

**Affiliations:** 1 Research Group Phytoplankton Community Interactions, Max Planck Institute for Chemical Ecology, Jena, Germany; 2 Research Group Mass Spectrometry/Proteomics, Max Planck Institute for Chemical Ecology, Jena, Germany; 3 Research Group Olfactory Coding, Department of Evolutionary Neuroethology, Max Planck Institute for Chemical Ecology, Jena, Germany; 4 Institute for Inorganic and Analytical Chemistry, Friedrich Schiller University Jena, Germany; 5 Max Planck Institute for Chemical Ecology, Jena, Germany; 6 Microverse Cluster, Friedrich Schiller University Jena, Germany

**Keywords:** algae, AP-SMALDI, ectoine, holobiont, high-resolution mass spectrometry, mass spectrometry imaging, marine bacteria, Ulva

## Abstract

Symbiosis is a dominant form of life that has been observed numerous times in marine ecosystems. For example, macroalgae coexist with bacteria that produce factors that promote algal growth and morphogenesis. The green macroalga *Ulva mutabilis* (Chlorophyta) develops into a callus-like phenotype in the absence of its essential bacterial symbionts *Roseovarius* sp. MS2 and *Maribacter* sp. MS6. Spatially resolved studies are required to understand symbiont interactions at the microscale level. Therefore, we used mass spectrometry profiling and imaging techniques with high spatial resolution and sensitivity to gain a new perspective on the mutualistic interactions between bacteria and macroalgae. Using atmospheric pressure scanning microprobe matrix-assisted laser desorption/ionisation high-resolution mass spectrometry (AP-SMALDI-HRMS), low-molecular-weight polar compounds were identified by comparative metabolomics in the chemosphere of *Ulva*. Choline (2-hydroxy-*N*,*N*,*N*-trimethylethan-1-aminium) was only determined in the alga grown under axenic conditions, whereas ectoine (1,4,5,6-tetrahydro-2-methyl-4-pyrimidinecarboxylic acid) was found in bacterial presence. Ectoine was used as a metabolic marker for localisation studies of *Roseovarius* sp. within the tripartite community because it was produced exclusively by these bacteria. By combining confocal laser scanning microscopy (cLSM) and AP-SMALDI-HRMS, we proved that *Roseovarius* sp. MS2 settled mainly in the rhizoidal zone (holdfast) of *U. mutabilis*. Our findings provide the fundament to decipher bacterial symbioses with multicellular hosts in aquatic ecosystems in an ecologically relevant context. As a versatile tool for microbiome research, the combined AP-SMALDI and cLSM imaging analysis with a resolution to level of a single bacterial cell can be easily applied to other microbial consortia and their hosts. The novelty of this contribution is the use of an in situ setup designed to avoid all types of external contamination and interferences while resolving spatial distributions of metabolites and identifying specific symbiotic bacteria.

## Introduction

In intertidal zones with high temporal and spatial ecosystem variations, bacteria and macroalgae establish close mutualistic relationships, in which both gain reciprocal benefits forming an ecological unit (holobiont) [1–3]. Chemical exchange and physical proximity are the basis of this algae–bacterial mutualism [4], but little is known about the spatial distribution of the bacteria on the algal host and the locally released and exchanged compounds within the algal chemosphere [3]. Bacterial biofilms on macroalgae can be crucial for developing algae and their interactions with other marine organisms. The exchange of resources in this spatially limited region is of high interest for understanding the macroalgal–bacterial interactions. The chemosphere was proposed as a region that supports chemical mediator-based cross-kingdom interactions [3]. High-throughput sequencing analysis provides the abundance and composition of the bacterial community on macroalgal surfaces [5–6]. It does not reveal any information on the metabolically active bacteria and the spatial distribution of substances exchanged. While the study of bacterial symbiosis is often limited to either chemistry or microscopy work, recent functional and metabolomics methods are available to enable chemical imaging of specialised metabolites involved in host–bacteria interactions.

In our study, comparative metabolomics using atmospheric pressure scanning microprobe matrix-assisted laser desorption/ionisation high-resolution mass spectrometry (AP-SMALDI-HRMS) enables the identification of specialised metabolites of the marine macroalga *Ulva mutabilis* (Chlorophyta) and its associated essential bacteria, a model system for cross-kingdom interactions [7]. The method provides a tool to formulate hypotheses about metabolic processes in the phycosphere while preserving spatial structure. This novel depth of insight into a multicellular host and bacteria interactions can characterise natural products in symbiotic interactions.

**Figure 1:**
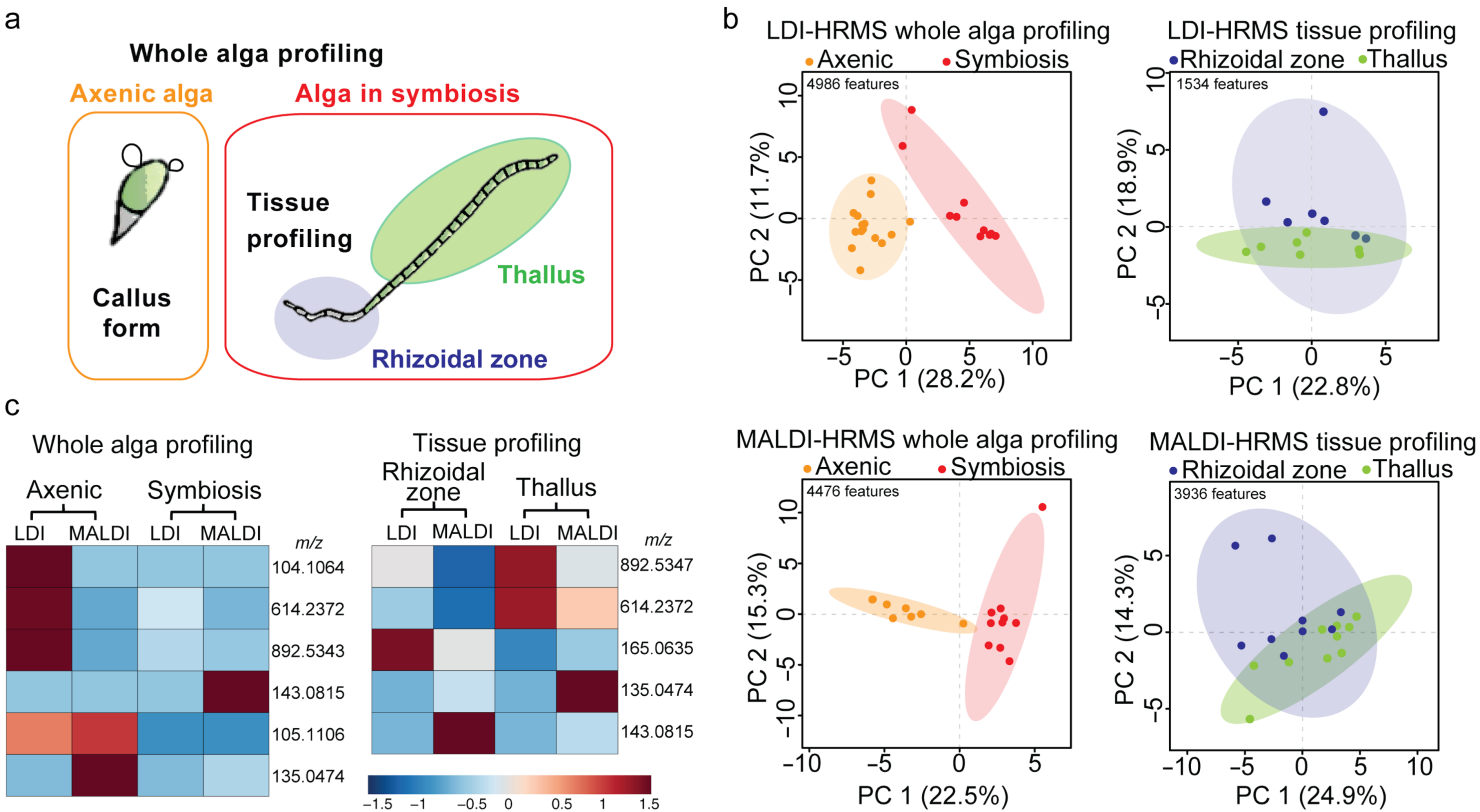
Untargeted comparative metabolomics using AP-SMALDI-HRMS highlighted metabolites involved in *Ulva*–bacteria symbiosis. a) The study looked at axenic algae with cell wall protrusions, the whole algae, and specific tissues with bacterial symbionts. b) The profiles of axenic alga (“axenic”) were contrasted with alga with bacterial symbionts (“symbiosis”) in the PCA score plots for LDI and MALDI-HRMS. The ellipses represent the 95% confidence region. c) The significant features (*m*/*z*) characterising axenic algae, algae in symbiosis, or differentiated tissues (blades/thalli, rhizoids) are represented in a heatmap with their relative abundance. The colour scale represents the averaged TIC normalised intensities per sample class (red colour for high intensity, blue for low intensity).

Algal growth and morphogenesis-promoting factors (AGMPFs) are required for the development of the model organism *U. mutabilis* [7]. They are provided by a combination of two essential bacteria, *Maribacter* sp. MS6 and *Roseovarius* sp. MS2 forming a tripartite community [3,7–8] (see also Figure 1a and the Graphical Abstract). In turn, *Roseovarius* sp. benefits from the released photosynthate glycerol as a carbon source [9]. Axenic *Ulva* germ cells (i.e. gametes) develop into a callus-like phenotype composed of undifferentiated cells with malformed cell walls [8,10]. Up to now, the bacterial sesquiterpenoid thallusin, released by *Maribacter* spp. [11–12], is the only known AGMPF that induces morphogenesis such as rhizoid and cell-wall formation in *Ulva* spp. [11–12] or thallus development in *Monostroma* spp. [13]. The *Roseovarius*-factor that promotes cell division is still unknown [3,8]. Algal substances are released into the surrounding environment to attract epiphytic bacteria and initiate the cross-kingdom interaction [14–15]. *Ulva* attracts *Roseovarius* sp. MS2 through the sulphur-containing zwitterion dimethylsulphoniopropionate (DMSP), resulting in biofilm formation on the algal surrounding [9]. The bacterium subsequently uses the provided glycerol for growth and transforms DMSP into methanethiol and dimethyl sulphide [9].

The metabolic activities of marine bacteria and algae can be surveyed using mass spectrometry-based methods. For example, stable sulphur isotope (^34^S) labelled DMSP was used to track DMSP uptake and degradation by marine bacteria, and secondary ion mass spectrometry was applied to visualise it at the single-cell level [16]. The interaction between epibiotic bacteria on algal surfaces and their metabolic activities can be monitored in situ or using an imprinting method by desorption electrospray ionisation mass spectrometry [17–18]. In *U. mutabilis* gametophytes, matrix-assisted laser desorption ionisation mass spectrometry imaging (MALDI-MSI) was used to identify cell differentiation markers [19]. However, there has yet to be a thorough investigation of associated-mutualistic bacteria. MALDI-MSI has been shown to have high sensitivity and spatial resolution at the microscale in plant tissues, plankton, and other microbes [20–21].

The application of a MALDI matrix to a sample is an important part of the MALDI-MSI experiment. MALDI-MS can be used to identify proteins and metabolic signatures [22–24] from bacteria and microalgae, as well as biofilms [25]. The primary function of the applied matrix is to improve the quality of the MS spectra, particularly the signal intensities of the compounds of interest. In some cases, the matrix might also work in opposition to this premise, suppressing desired ions. Then, matrix-free approaches such as LDI-HRMS can overcome this limiting phenomenon and have been applied for species-level microalgal identification based on metabolic profile fingerprint matching [26–28].

Our research combines cutting-edge laser scanning microscopy and high-resolution mass spectrometry to uncover *Ulva*/bacteria interactions and specialised metabolites at the microscale level. In this study, we demonstrate that the chemosphere of *U. mutabilis* changes depending on the presence or absence of the bacterial symbionts (*Roseovarius* sp. MS2 and *Maribacter* sp. MS6). As a result, specific metabolic markers can be used to identify bacteria in the vicinity of *U. mutabilis*. We used an untargeted comparative metabolomics approach that also provides micrometre-resolved MS imaging data through AP-SMALDI-HRMS. Two sample preparations, matrix-free LDI and MALDI, were performed to increase the range of metabolites recovered with this type of ionisation. We identified significant metabolites that define the host–bacteria interactions based on spectral similarity with standards. Using combined imaging mass spectrometry and confocal laser scanning microscopy, we then linked the chemical and microscopic observations that characterise the symbiotic association (cLSM).

## Results and Discussion

### Comparative metabolomics using AP-SMALDI-HRMS identifies metabolites in axenic algae and those present during macroalgal–bacterial symbiosis

Axenic gametes of *U. mutabilis* (phenotype slender) were allowed to settle onto glass plates in Petri dishes filled with growth medium. In the absence of the symbionts, the axenic gametes developed into undifferentiated cells known as the callus-like form [8,29]. In the second set of samples, algae were inoculated with the two marine bacteria, *Roseovarius* sp. MS2 and *Maribacter* sp. MS6, developing into a phenotype composed of bilayer cells and organised tissues, as previously reported [8]. The algal germlings incubated with the marine bacteria showed a rhizoidal zone that serves for substrate attachment and a thallus zone. From apex to rhizoid, *Ulva* germlings had an average length of 50 to 150 µm after three weeks of growth. The samples were recovered, dried on tissue, and for MALDI, immediately covered with 2,5-dihydroxybenzoic acid (DHB) applied by spraying. We targeted either specialised tissues (rhizoidal zone versus thallus) or the whole alga germlings (axenic callus versus alga in symbiosis) using a mounted AP-SMALDI camera (Figure 1a). The metabolic profiles of tissue and whole alga were obtained from callus or alga in symbiosis using AP-SMALDI-HRMS with the two sample preparations, either with matrix deposition (MALDI-HRMS) or matrix-free analysis (LDI-HRMS) (Figure 1a and b). The data matrix was generated by processing the raw spectra, and the data tables produced were from 1534 to 4986 features (*m*/*z*) (Figure 1b and Table S1 in Supporting Information File 1). The principal component analysis (PCA) visualised differences between metabolic profiles of axenic algae, algae in symbiosis, and specialised tissues (thallus, rhizoidal zone), analysed either with LDI or MALDI-HRMS. The metabolic profiles of axenic algae and algae in symbiosis were significantly different, while tissue-specialised metabolomes were less differentiated in the PCA score plots (Figure 1b). Significant features in the loading plots were listed in a heatmap to compare their relative abundance of intensities averaged per sample class (Figure 1c). Among the statistically significant features in all datasets (Table S1, Supporting Information File 1), six metabolites were identified, which were annotated using spectral similarity with analytical standards. For example, the features *m*/*z* 104.1064 and *m*/*z* 143.0815 were selected among the significant markers of the MALDI-HRMS profiling of axenic algae and the rhizoid tissue (whole alga profiling) of *U. mutabilis* grown with the marine symbiotic bacteria, respectively (Figure 1c). The heatmap shows the complementarities of both methods, LDI or MALDI-HRMS, as the significant features *m*/*z* 104.1064 and *m*/*z* 143.0815 have only been detected by one of the two methods.

### Identification of metabolites in *Ulva*–bacteria symbiosis

**Figure 2:**
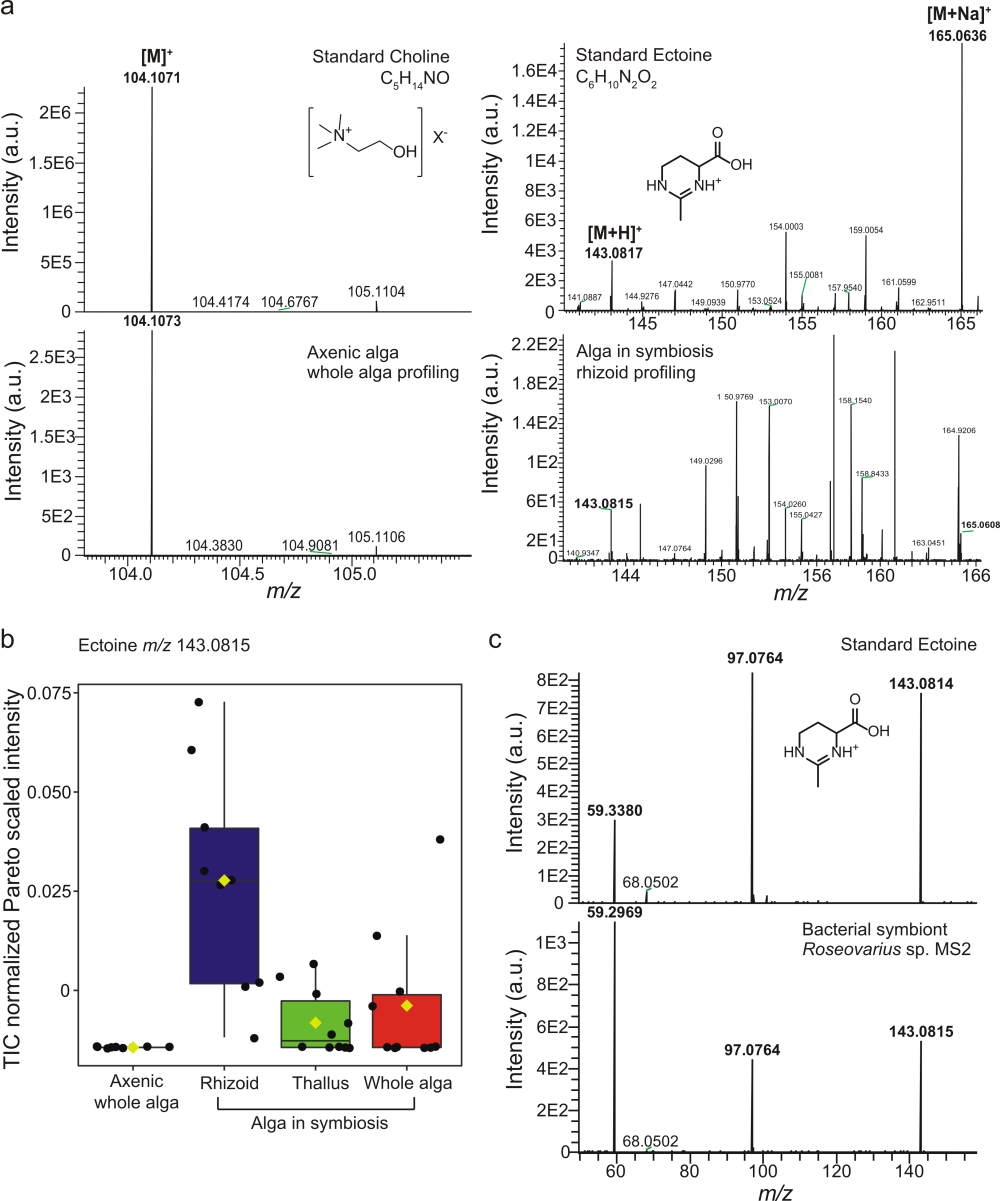
Identification of significant features associated with axenic or bacterial symbiont-associated alga *Ulva mutabilis* (phenotype slender). a) The structural determination was achieved by spectral matching with the analytical standards using AP-SMALDI-HRMS. b) Relative amounts of ectoine (*m*/*z* 143.0815 for [M + H]^+^) were determined by AP-SMALDI-HRMS measurements to compare different tissues: axenic and algae in symbiosis. One-way ANOVA with a Fisher HSD post hoc test found choline to be significant in profiles of axenic algae (F = 42, *P*-value < 0.0001) and ectoine in profiles of rhizoidal zones of algae in symbiosis (F = 4, *P*-value < 0.005) (colour code with reference to Figure 1a). c) Ectoine (*m*/*z* 143.0815 for [M + H]^+^, precursor ion) was identified in a single colony of the bacterial symbiont *Roseovarius* sp. MS2 using AP-SMALDI-HRMS/MS analysis.

To identify the selected markers found by the comparative metabolomics study, we searched several mass spectra libraries, including METLIN, and determined the chemical formula based on exact mass. We also used spectral similarity matching of data acquired from analytical standards. Choline was identified from the molecular peak *m*/*z* 104.1064 for [M]^+^ (calculated *m*/*z* as 104.1069 ± 4.8 ppm for C_5_H_14_NO) in the profiles of axenic *U. mutabilis* (Figure 2a). This small polar metabolite was linked to the metabolic homeostasis of *Ulva lactuca* during tidal cycles [30]. Choline is the precursor of the membrane constituent phosphatidylcholine [31]. We inferred that the accumulation of choline in axenic *U. mutabilis* germlings might correlate with the absence of the key bacterial morphogen thallusin, which induces cell wall and rhizoid formation. The accompanying formation of cell wall protrusions might disrupt the cell membrane arrangement indicated by choline accumulation. Screening the tripartite community *Ulva–Roseovarius–Maribacter* identified ectoine as a metabolic marker of the rhizoidal zone (Figure 2b). The molecular formula C_6_H_10_N_2_O_2_ was deduced from the molecular peak at *m*/*z* 143.0817 for [M + H]^+^ (± 1.4 ppm) and *m*/*z* 165.0636 for [M + Na]^+^ (± 1.2 ppm) detected in the AP-SMALDI-HRMS profiles of the standard and rhizoid tissue of *U. mutabilis* in symbiosis with the marine bacteria. To separate algal and bacterial metabolism, single colonies of *Roseovarius* sp. MS2 and *Maribacter* sp. MS6 were deposited onto glass slides and analysed with AP-SMALDI-HRMS/MS. Using spectral similarity matching based on the fragmentation pattern obtained from AP-SMALDI-HRMS/MS experiments, we proved that the bacterial symbiont *Roseovarius* sp. MS2 produces ectoine (Figure 2c). This observation supports earlier assumptions that the rhizoidal zone is mainly colonised by *Roseovarius* sp. MS2 [8,29].

Ectoine is a known osmoprotectant produced by marine bacteria and phytoplankton with high concentrations during saline stress conditions [32]. It has not yet been described in the *Ulva*–bacteria symbiosis. Not all essential genes for ectoine biosynthesis reported by [33] were found in the *U. mutabilis* genome [34], providing further support for the bacterial origin of ectoine. Homologs of EctA (UM017_0070.1, E value 0.34), EctB (UM084_0040.1, E value < 0.0001) that provide the central intermediate *N*-acetyl-2,4-diaminobutyrate and EctD (UM025_0127.1, E value 0.094) an ectoine hydroxylase could be identified. However, a homolog gene for EctC (ectoine synthase) is missing in the *U. mutabilis* genome. In addition, despite the low E value of EctB, the reciprocal NCBI-blast search against the anoxygenic photosynthetic halophile and ectoine-producing bacterium *Halorhodospira halochloris* [35] did not confirm the presence of the sequence in the algal genome. Therefore, it is unlikely that the alga produces ectoine. In summary, ectoine is indicative of *Roseovarius* sp. MS2 in the tripartite community and can serve for localisation studies.

### Localisation of bacterial symbionts of *Ulva mutabilis* using fluorescence microscopy and imaging mass spectrometry

**Figure 3:**
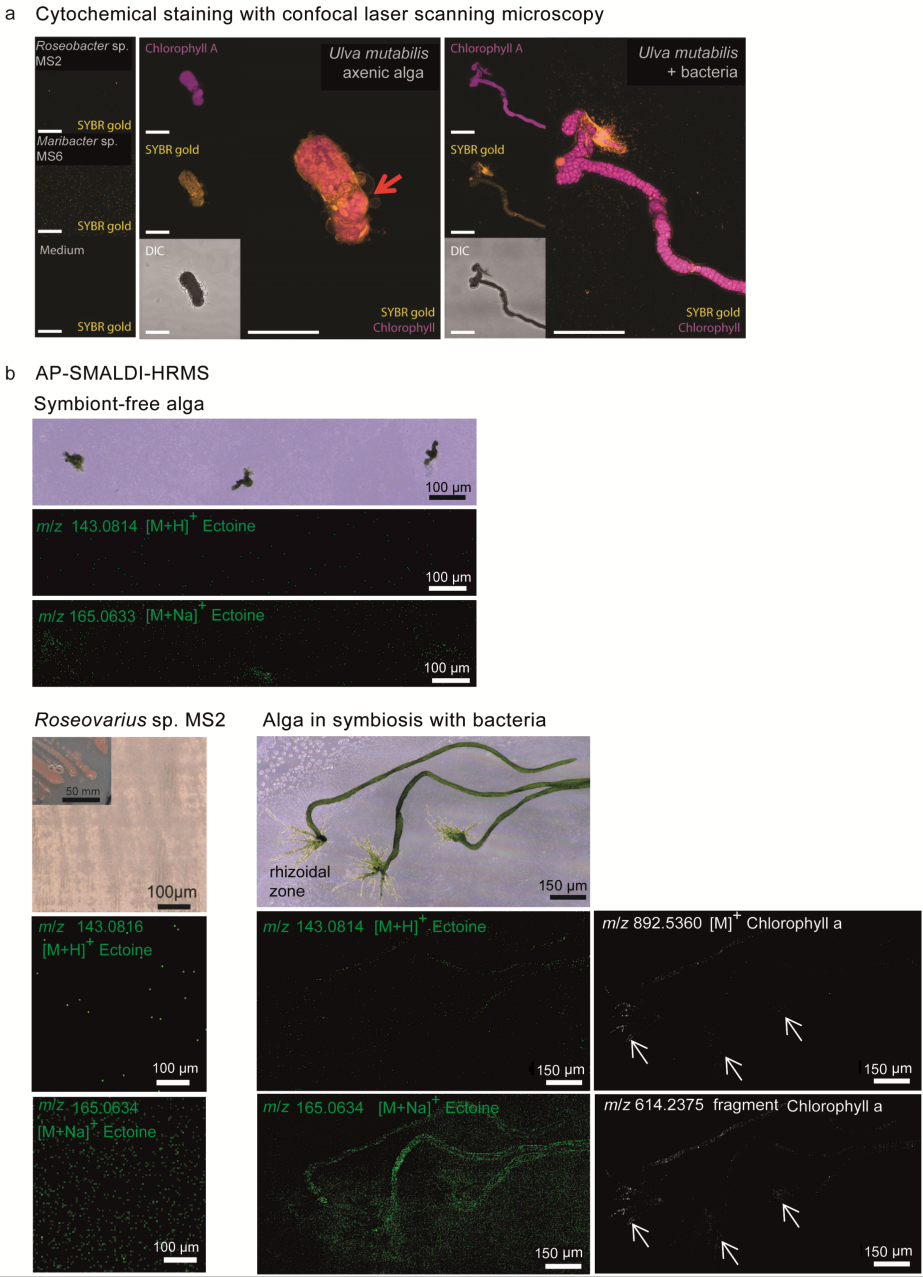
Visualisation of algae *Ulva mutabilis* grown under axenic conditions or with bacterial symbionts *Roseovarius* sp. MS2 and *Maribacter* sp. MS6. a) Images acquired after nucleic acid staining with SYBR gold and with confocal laser scanning microscopy. The protrusion of alga grown without bacterial symbiont is highlighted (red arrow). DIC: differential interference microscopy. b) The images show ectoine spatial localisation and thus the presence of *Roseovarius* sp. (*m*/*z* 143.0814 for [M + H]^+^, *m*/*z* 165.0633 for [M + Na]^+^, shown in green) as well as chlorophyll (*m*/*z* 892.5360 for [M + H]^+^, *m*/*z* 614.2375 fragment shown in white). These metabolite traces are visible in axenic algae, symbiotic algae, and cell cultures of bacteria *Roseovarius* sp. MS2. White arrows indicate the rhizoidal zones.

Based on the above results, we combined LDI-MS imaging mass spectrometry and cLSM using a non-specific fluorescence labelling probe to visualise the bacterial cells living in symbiosis with *U. mutabilis*. Following a one-month incubation in clean cuvette slides placed in Petri dishes filled with medium, axenic and bacteria-inoculated *U. mutabilis* germlings were stained with SYBR Gold, a sensitive probe forming a complex with DNA with high fluorescence quantum yield [36]. In the axenic callus-like form, the nuclei of algal cells and the bacterial cells accumulated around the rhizoidal tissue and exhibited the specific fluorescence after SYBR Gold staining (Figure 3a) as previously described [8,37]. These findings indicated that bacteria are associated with their algal host during symbiosis.

In parallel, we visualised the metabolites produced by the biofilm formed around *U. mutabilis* by imaging analysis with AP-SMALDI-HRMS. Three replicates each of the axenic algae, algae in symbiosis, germlings, and bacterial cells in monocultures were imaged after matrix deposition by AP-SMALDI-HRMS over a centimetre-scaled area (Figure 3b). The algal pigment chlorophyll was localised with the algal tissues (Figure 3b and Figure S1 in Supporting Information File 1). Even though most of the seawater media was removed from the *Ulva* samples during sample preparation, crystallisation of seawater salts on the sample surface occurred. The size of the crystals and their distribution within an imaged area were examined using a digital microscope and found to be homogeneous and consistent across the samples and experiments. As a result, the ion suppression effect caused by the presence of seawater crystals on the *Ulva* samples and surroundings was consistent across all measurements (Supporting Information File 1, Figures S1 and S2).

Ectoine was detected in both profiling and imaging MS spectra as the [M + Na]^+^ adduct at *m*/*z* 165.0633. Ectoine was mainly found around the rhizoid in elevated amounts. Thus, *Roseovarius* sp. MS2 became visible in the rhizoidal zone and on the thallus due to the exclusive production of ectoine within the tripartite community (Figure 3b). AP-SMALDI-HRMS studies extended to the entire clade of motile Rhodobacteraceae will reveal other characteristic metabolites of the *Ulva*–bacteria interactions. Those species attracted by *U. mutabilis* (e.g., through DMSP) that use the provided photosynthates [9], will preferentially succeed the previously described competitive colonisations of *Ulva* spp. [38–39]. Also, related species of *Roseovarius* sp. MS2 can often release unknown AGMPFs [29,40], which further foster the bacterial–algal interactions. As more species-specific metabolic markers become available, AP-SMALDI imaging will be a powerful tool to track these dynamic microbial colonisation processes using the *U. mutabilis* model system with a designed microbiome.

## Conclusion

Metabolic profiling of whole alga and specialised tissues conducted with AP-SMALDI-HRMS enabled identifying specific metabolites in host–bacteria symbiosis. We report the first identification of choline and ectoine as markers of symbiont-free *U. mutabilis* and rhizoid tissue of algae in symbiosis with bacteria. We visualised the rhizoidal zone formed by the bacterial symbionts using chemical staining, confocal laser scanning microscopy, and imaging mass spectrometry. Notably, ectoine was used as a metabolic marker to identify bacteria in the biofilm associated with *U. mutabilis* and the algal surface. Visualising the spatial distribution of epiphytic bacteria in the phycosphere will contribute to the general understanding of the chemically mediated cross-kingdom interactions. The combined AP-SMALDI and cLSM imaging with resolution down to the level of a single bacterial cell introduced here can be applied to other microbial consortia and their hosts and will be instrumental for microbiome research.

## Experimental

### Biological experiments and imaging microscopy

The laboratory strains of *U. mutabilis* (sl-G[mt+]) are direct descendants of the original isolates collected by B. Føyn in Portugal (Ria Formosa) in 1958 [8]. This strain is used as a model organism in cross-kingdom interactions [7,34,37] and cultivated under standardised conditions [41–42]. *Ulva* strains are available from the corresponding author (Thomas Wichard, Friedrich-Schiller-Universität Jena, Germany).

Gametogenesis of *U. mutabilis* was induced by chopping harvested algal tissue, and released gametes were purified from accompanying bacteria according to the protocol of Wichard and Oertel (2010) [41]. The strains *Roseovarius* sp. MS2 (Genbank EU359909) and *Maribacter* sp. MS6 (Genbank EU359911) were originally isolated from *U. mutabilis* [8] and were cultivated in Marine Broth medium (Roth, Germany) at 20 °C. *Ulva* gametes were either grown axenically or inoculated with the bacteria (final optical density OD_620_ = 0.001). All algae were cultivated in *Ulva* culture medium (UCM) [43] at 18 °C with the illumination of about 60 μmol photons m^−2^ s^−1^ under a 17:7 light/dark regime. Axenic *Ulva* gametes deposited on cleaned glass slides and inoculated with bacteria MS2/MS6 were prepared following the procedure for in situ MS imaging described by Kessler et al. [19]. Briefly, algal gametes were inoculated to 10 mL medium in 9 cm diameter sterile Petri dishes with a clean and autoclaved glass slide (25 mm × 75 mm) with cavities (Paul Marienfeld, Germany) on the bottom; samples were incubated for one month at 18 °C in static conditions. An inverted microscope was used to monitor the algal growth. Transmitted light microscopy pictures were obtained using a Keyence BHX-500 digital microscope. Samples were recovered with pliers and fixed with glutaraldehyde 1% (Merck), stained with SYBR Gold (1% in DMSO, Invitrogen, Thermo Fisher); a cover slide was added, followed by incubation in the dark at 15 °C for 15 min. Cavity slides were spotted with 100 µL of SYBR Gold or unstained bacterial monoculture (*Roseovarius* sp MS2 or *Maribacter* sp. MS6) to use them as controls. Fluorescence images (1024 × 1024) were acquired using a Zeiss cLSM 880 (Carl Zeiss AG, Oberkochen, Germany) with a Plan-Apochromat 20 × 0.8 and 488 nm Argon-laser excitation (5% transmission). Emission wavelengths for SYBR Gold (490–650 nm) and chlorophyll A (653–735 nm) were separated via the spectral detection unit. Transmitted light was detected by the transmitted light-PMT. The effect of an additional quick washing step was tested by gently adding 100 µL of sterile MQ water for two seconds. The controls consisted of bacteria grown for one week in monoculture in 40 mL of marine broth medium and the axenic medium with fixative and stain. All the experiments with glass slides were performed in biological triplicates.

### Genome analysis

To identify the putative biosynthetic gene cluster (*ect* gene cluster) in *U. mutabilis* [34], the algal genome was searched for the gene ectoine hydroxylase (*ectD*) and also for a specialised aspartokinase (*ask_ect*). Aspartokinase (Ask), along with ʟ-aspartate-β-semialdehyde-dehydrogenase (Asd), provides the precursor ʟ-ASA for ectoine biosynthesis [33,44–45]. Homologs of the enzymes of the ectoine pathway from *Halorhodospira halochloris* were identified by BLAST searches of the *U. mutabilis* genome at ORCAE using default parameters (https://bioinformatics.psb.ugent.be/orcae/overview/Ulvmu).

### AP-SMALDI-HRMS metabolic profiling and imaging

All standards and *Ulva* samples were analysed via AP-SMALDI (AP-SMALDI10, TransMit, Germany) ion source equipped with a UV (337 nm) nitrogen laser (LTB MNL-106, LTB, Germany) coupled to a high-resolution mass spectrometer Q-Exactive Plus (Thermo Fisher Scientific, Bremen, Germany). Glass slides with one month-grown algal gametophytes were gently recovered from a Petri dish filled with UCM using a sterile tweezer and dipped for one second in sterile ultrapure water to remove the excess salts before metabolic profiling. When algae were investigated directly on a glass slide before in situ MS imaging, blotting paper was used to remove sea water (see also Supporting Information File 1). The desired area of a glass slide covered with algal individuals was first marked, photographed, and finally fixed on the AP-SMALDI metal target.

AP-SMALDI profiling and imaging experiments unless otherwise stated were enhanced by a 2,5-dihydroxybenzoic acid (DHB) MALDI matrix. A methanolic solution of the DHB matrix at a concentration of 4 mg mL^−1^ was applied onto a sample via SunChrom MALDI spotter (SunChrom GmbH, Germany). The spraying method was optimised using the following parameters: line distance 2 mm, spraying speed 800 mm min^−1^ with 5 seconds drying time, and matrix solution flow rate in 4 cycles from 10 μL up to 30 μL min^−1^. Solvents used in this study were all LCMS analytical grade. 2,5-Dihydroxybenzoic acid with a purity of above 98% and high purity MS-grade methanol were purchased from Sigma-Aldrich (Germany).

All *Ulva* samples were imaged in the positive ion mode using a step size of 5 μm and with the number of laser shots per spot set to 30 (approximately 1.2 μJ shot^−1^) within the laser frequency of 60 Hz. MS spectra were acquired in a mass range from *m/z* 100 to *m/z* 1000 with a resolving power of 280000. Pseudo ion intensity maps of selected *m*/*z* values were generated using the Mirion V3 software package with an *m*/*z* width of 0.01 u.

In the profiling mode, the single *Ulva* individuals were targeted visually and ablated with a laser spot size of approximately 10 μm in positive and negative polarity in a mass range from *m/z* 100 to *m/z* 1000. The other parameters stayed like for the MSI mode. In profiling, the same area of the rhizoid and the tip of the thallus of different individuals were analysed by laser ablation over one-minute time acquisition. Axenic and alga in symbiosis germlings were profiled with a UV laser along a longitudinal axis to investigate the effect of bacteria on metabolism changes in *U. mutabilis*.

The size of the sample groups analysed by AP-SMALDI-HRMS in profiling mode was *n* = 10 for thallus tissue, *n* = 9 for rhizoid tissue, *n* = 8 for axenic callus, and *n* = 10 for alga in symbiosis. Matrix-free experiments (LDI-HRMS) were performed in profiling mode under the same experimental conditions as the AP-SMALDI-HRMS. The size of the sample groups was defined as follows: *n* = 6 for rhizoid, *n* = 7 for thallus and whole alga profiling, *n* = 10 for alga in symbiosis, and *n* = 15 for axenic alga.

The metabolic profiles of nutrient media were obtained by analysing 30 µL deposited onto cleaned glass slides and following the same protocol used for the *Ulva* samples. In the late exponential stage, bacterial monoculture was recovered from agar plates with a 10 µL loop and diluted in 100 µL of sterile water. Five microliters of the solution were spotted onto a glass slide and analysed in AP-SMALDI-HRMS mode in positive and negative polarity.

The data acquired in MSI mode were collected with Xcalibur software version 2.8 SP1 Build 2806 (Thermo Fisher Scientific, Germany) while the acquisition of spatial scans, pre-defined in the *x*- and *y*-direction as rectangular sample regions, was controlled by the MCP (Master Control Program, TransMIT GmbH, Giessen, Germany). The raw data acquired in profiling mode were visualised in Thermo Xcalibur™ version 3.0.63 (Thermo Fisher Scientific, Germany) and then converted to netCDF format using the Thermo File Converter tool. Data pre-processing was performed to extract the intensities in each profile, excluding the features of the nutrient medium using a script adapted from the MALDIquand package [46]. Spectra were de-noised with a signal-to-noise ratio of 5. Normalisation was done based on total ion current (TIC) recommended for MALDI-MS analysis [47]. All spectra, images, R data, scripts, and results from the statistical analysis were uploaded and are freely accessible in the Max Planck repository Edmond (https://dx.doi.org/10.17617/3.4v).

### Significant features analysis and metabolite identification

Data analysis was conducted in MetaboAnalyst 4.0 [48] to perform univariate and multivariate statistical tests and find significant differences in intensities and the presence or absence of metabolites in the samples. Pareto scaling and cube root transformation were conducted to normalise the datasets before the multivariate statistics. PCA highlighted the metabolic differences between axenic and alga in symbiosis and between thallus and rhizoid tissues. Significant features were searched in the PCA loading plots and also in the pattern hunter plots obtained from a correlation analysis based on the Pearson correlation coefficient R. A one-way ANOVA with Fisher's LSD post hoc test (*P*-value < 0.05) was performed, and the relative amounts of the significant features were displayed as a boxplot. The selected significant features were further searched in the raw HRMS profiles to identify those with the reliable isotopic pattern assigned to a metabolite. The *m*/*z* values were searched in the METLIN database, using a mass deviation equal to or lower than five ppm, which suggested several known natural products such as ectoine [49].

To confirm the identity of the significant features, mass spectral information was compared with analytical standards analysed with the AP-SMALDI-HRMS (DMSP, chlorophyll-a, ectoine, choline). MS/MS experiments were performed with AP-SMALDI-HRMS to match the fragmentation pattern between the standard ectoine and bacteria monoculture profile. Fragmentation spectra of ectoine were acquired from the bacterial isolate *Roseovarius* sp. MS2 and an ectoine standard. To perform a measurement, 4 μL of ectoine at concentration 50 μM was pipetted onto a clean glass slide (washed with dH_2_O, acetone) and overlaid with 2 μL of a methanolic solution of the DHB matrix at a concentration of 4 mg mL^−1^. For a bacterial isolate, the sample was prepared from one colony smeared onto a glass slide and covered with the DHB matrix, following the standard ectoine procedure. Samples were analysed in positive ion mode, with the number of laser shots per spot set to 30 (approximately 1.2 μJ shot^−1^). All-ion fragmentation (AIF) mode was set as follows: molecular ion of ectoine at *m*/*z* 143.1; isolation window *m*/*z* ± 0.2; 45 NCE. The peak resolution was set at 280000, and the mass range was set from *m*/*z* 50 to *m*/*z* 300.

## Supporting Information

File 1 Details on sample preparation and additional figures.
