# Supplementary material for: A new glance at the chemosphere of macroalgal–bacterial interactions: In situ profiling of metabolites in symbiosis by mass spectrometry
Source: Beilstein J Org Chem. 2021 May 19;17:1313–22. doi: 10.3762/bjoc.17.91 (PMC8182680; doi:10.3762/bjoc.17.91)
Supplement: File 1 — Details on sample preparation and additional figures. [file Beilstein_J_Org_Chem-17-1313-s001.pdf]

BEILSTEIN JOURNAL OF ORGANIC CHEMISTRY

Supporting Information

for

A new glance at the chemosphere of macroalgal–bacterial interactions: In situ profiling of metabolites in symbiosis by mass spectrometry

Marine Vallet, Filip Kaftan, Veit Grabe, Fatemeh Ghaderiardakani, Simona Fenizia, Aleš Svatoš, Georg Pohnert and Thomas Wichard

Beilstein J. Org. Chem. **2021,** *17,* 1313–1322. doi:10.3762/bjoc.17.91

Details on sample preparation and additional figures

License and Terms: This is a supporting information file under the terms of the Creative Commons Attribution License (https://creativecommons.org/licenses/by/4.0). Please note that the reuse, redistribution and reproduction in particular requires that the author(s) and source are credited and that individual graphics may be subject to special legal provisions.
The license is subject to the *Beilstein Journal of Organic Chemistry* terms and conditions: (https://www.beilstein-journals.org/bjoc/terms)

Content

Table S1 Descriptive information and significant features found S2

Notes on sample preparation methodology S3

Figure S1: The effect of seawater salt crystallisation...S4

Figure S2: AP-SMALDI HR MSI on a map of sodium adducts S5

Table S1: Descriptive information and significant features found by the Student's test in the LDI and MALDI-HRMS datasets for tissue profiling and whole alga profiling (compare with Figure 1b). The results of the Student's tests can be found on the Edmond – the Open Research Data Repository of the Max Planck Society. (Title: "High-resolution mass spectrometry-guided identification of metabolites in *Ulva*-bacteria symbiosis"; Max Planck Society, submitted by Marine Vallet, 2020; https://dx.doi.org/10.17617/3.4v)

Dataset	Samples number	Total number of features analyzed	Number of significant features found by Student's test (p-value < 0.05)	Number of significant features found by Student's test (p-value < 0.01)
LDI-HRMS tissue profiling: (rhizoidal zone *versus* thallus)	14	1534	653	66
LDI-HRMS whole alga profiling: (axenic culture *versus* symbiosis)	25	4986	1399	970
MALDI-HRMS tissue profiling: (rhizoidal zone *versus* thallus)	19	3936	290	86
MALDI-HRMS whole alga profiling: (axenic culture *versus* symbiosis)	18	4476	676	327

Notes on the sample preparation methodology: Excess seawater salts were removed from the glass slide substrate's surface with a fine blotting paper placed on the side of the glass slide while the medium was absorbed. This simple method was successful in removing most of the diluted salts without disrupting the actual sample. This sample preparation step ensured that the seawater crystals were distributed almost uniformly across the imaged area.

Ion maps of potassium ($[M + K]^+$) and sodium adduct ($[M + Na]^+$) of the compound(s) with the molecular formula $C_{10}H_{16}O_3$ were generated to show the presence of seawater salts and their distribution across the *Ulva* sample attached to a glass slide (Figure S1).

Ion maps confirmed that both cations were distributed uniformly throughout the imaged area, albeit at slightly different concentrations. A region covered with slightly larger crystals served better as a source of potassium cations, according to optical images taken with a digital microscope before and after UV laser ablation. Fine crystal-covered regions, on the other hand, appear to facilitate the formation of sodium adducts.

Based on the exact mass (error 0.5 ppm) and calculated best matching chemical formula and isotope simulation generated by Qual Browser/Xcalibur software 3.0.63, ions at *m/z* 207.0991 ($[M + Na]^+$) and *m/z* 223.0731 ($[M + K]^+$) were assigned to $C_{10}H_{16}O_3$.

The 2D ion map of the sodium adduct of ectoine at *m*/*z* 165.0634 ($[M + Na]^+$) is completely consistent with the interpretation of sodium cation distribution within an imaged area (Figure S2).

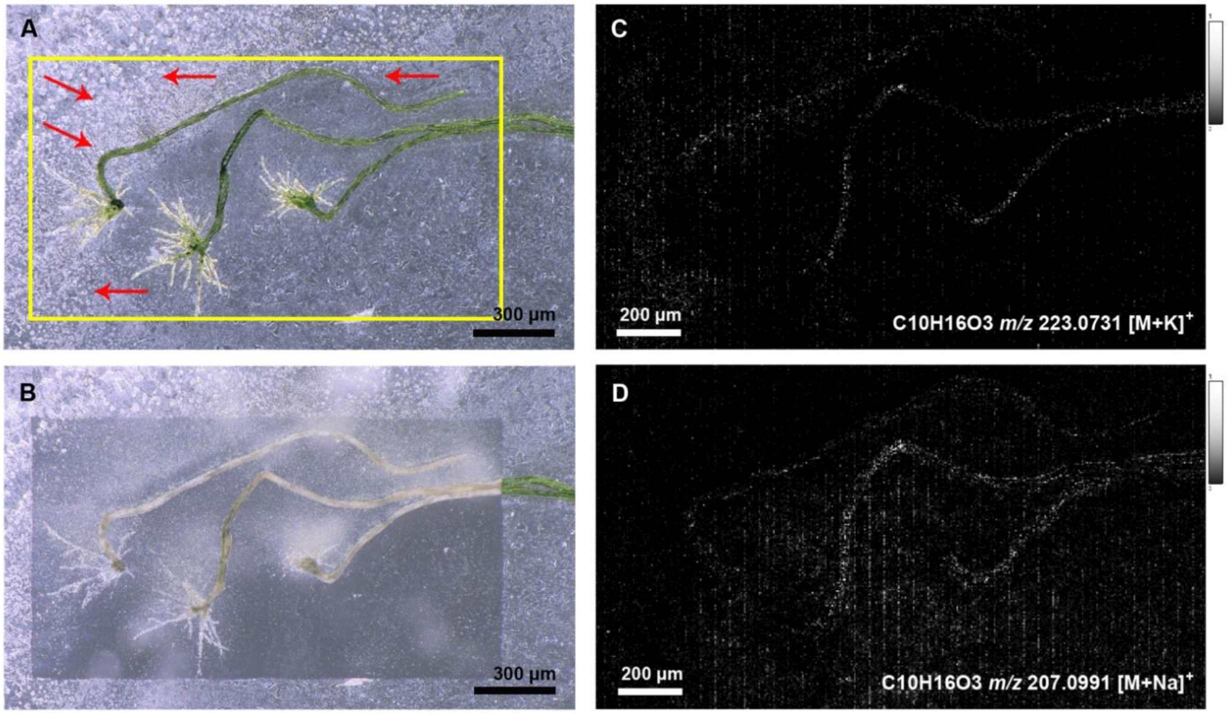

Figure S1: The effect of seawater salt crystallisation on the distribution of sodium and potassium cations on a glass substrate. Panel (A) optical image of a dry *Ulva* germling sample attached to a glass slide prior to the AP-SMALDI-HR MSI experiment, with arrows pointing to regions with thicker salt coating; (B) *Ulva* germling samples after the AP-SMALDI-HR MSI experiment; (C) ion map representing a potassium adduct of $C_{10}H_{16}O_3$ at *m/z* 223.0731 acquired during the AP-SMALDI-HR MSI experiment; (D) ion map representing a sodium adduct of $C_{10}H_{16}O_3$ at *m/z* 207.0991 acquired during AP-SMALDI-HR MSI experiment.

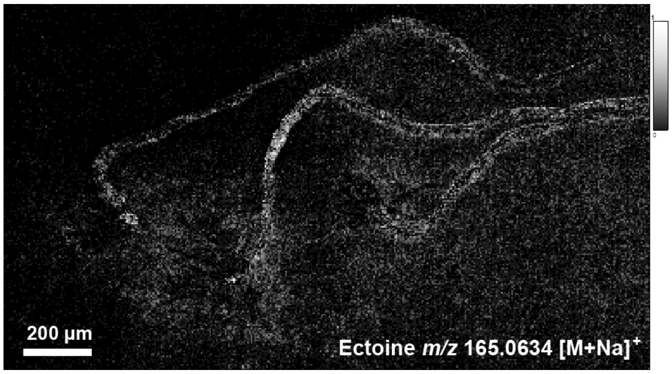

Figure S2: AP-SMALDI-HRMS on a ion map of sodium adduct of ectoine at *m/z* 165.0634, indicating *Roseovarius* sp. in the biofilm and on the *Ulva* algal surface of the germling.
